# Gorlin syndrome presenting with a unilateral ovarian fibroma in a 22-year-old woman: a case report

**DOI:** 10.1186/1752-1947-6-148

**Published:** 2012-06-12

**Authors:** Terence Finch, Chitra Pushpanathan, Krista Brown, Yasser El-Gohary

**Affiliations:** 1 Discipline of Laboratory Medicine, Faculty of Medicine, Memorial University, 300 Prince Philip Drive, St John’s, NL, A1B 3V6, Canada; 2 Janeway Children's Hospital and Rehabilitation Centre, Faculty of Medicine, Memorial University, 300 Prince Philip Drive, St John’s, NL, A1B 3V6, Canada; 3 Discipline of Obstetrics and Gynecology, Faculty of Medicine, Memorial University, 300 Prince Philip Drive, St John’s, NL, A1B 3V6, Canada

## Abstract

****Introduction**:**

Nevoid basal cell carcinoma syndrome, or Gorlin syndrome, is an inherited disorder characterized by malignancies of the skin and other organs, skeletal abnormalities, and congenital malformations. The syndrome follows an autosomal dominant inheritance pattern with a gene mutation localized to 9q22.3.

****Case presentation**:**

We present the case of a 22-year-old Caucasian woman with a unilateral ovarian fibroma, falx cerebri calcification and odontogenic keratocysts, but without any skin manifestations. The diagnosis of nevoid basal cell carcinoma syndrome was made after a right salpingo-oophorectomy for a calcified ovarian fibroma with cystic degeneration. Pathologic examination of the 10 cm right ovarian mass revealed a well-circumscribed spindle cell lesion. Immunohistochemical staining of the lesion demonstrated positivity for vimentin and smooth muscle actin.

****Conclusion**:**

It is important to recognize that nevoid basal cell carcinoma syndrome may present in the absence of skin lesions. Additionally, ovarian fibromas are typically bilateral in nevoid basal cell carcinoma syndrome, but can uncommonly be unilateral, which may alter clinical management. Ovarian fibromas are managed with surgical excision with an attempt at ovarian functional preservation.

## **Introduction**

Nevoid basal cell carcinoma syndrome, or Gorlin syndrome, is a rare hereditary multisystem disorder. It is characterized by a series of multiple developmental anomalies and increased risk of developing various benign and malignant tumors. Clinical manifestations of the disorder include odontogenic keratocysts of the jaw, macrocephaly, intracranial ectopic calcification (including calcified falx cerebri), palmer or plantar pits, and rib anomalies. Patients with Gorlin syndrome typically develop basal cell carcinoma at an early age, during adolescence or early adulthood. Various benign and malignant tumors may involve the skin, bone, genitourinary system and central nervous system. Epidermal cysts, ovarian fibromas, fibrosarcoma, cardiac fibroma, fetal rhabdomyosarcoma, meningioma and medulloblastoma have been reported. Numerous other anomalies and, less commonly, intellectual disability have also been observed [1].

The prevalence is estimated to be between one in 55,600 to 164,000 [2,3]. Germline mutations of the patched tumor suppressor gene, mapped to chromosome 9q22.3, are known to cause the syndrome [4,5]. The diagnosis can be made using the Kimonis *et al*. clinical diagnostic criteria, with two major or one major and two minor criteria:

Major criteria

Two or more basal-cell carcinomas, or one in person younger than 20 years

Odontogenic keratocysts of the jaw

Three or more palmar or plantar pits

Bilamellar calcification of the falx cerebri

Bifid, fused, or markedly splayed ribs

First-degree relative with Gorlin syndrome

Minor criteria

Macrocephaly

Congenital malformations such as cleft lip or palate, frontal bossing, coarse face, or hypertelorism

Other skeletal abnormalities such as Sprengel’s deformity, marked pectus deformity, or syndactyly

Radiologic abnormalities such as bridging of the sella turcica, vertebral anomalies (for example, hemivertebrae, fusion or elongation of the vertebral bodies), modeling defects of the hands and feet, or flame-shaped lucencies of the hands or feet

Ovarian fibroma

Medulloblastoma

Additionally, linkage analysis or polymerase chain reaction for direct mutation detection can be used [6]. Although the number of basal cell carcinoma lesions of the skin can be very high, it is important to note that the patient does not require multiple skin lesions for diagnosis, and that 10% of patients over the age of 30 years do not have these lesions [1,6].

The commonest gynecological manifestation is bilateral ovarian fibromas, however endometrial adenocarcinoma and ovarian fibrosarcoma have been described [1,7,8]. Ovarian fibromas are neoplasms of ovarian stromal cells which most commonly arise after puberty and constitute approximately 5% of ovarian neoplasms. Ovarian fibromas do not elaborate hormones and, in nonsyndromic cases, are usually unilateral with an average size of 6 cm. Cases of ovarian fibromas arising before puberty have been reported both in association with Gorlin syndrome and sporadically [8,9]. Ovarian fibromas were first reported to occur in conjunction with Gorlin syndrome in 1963 [8]. Syndromic fibromas are most often bilateral and calcified [1]. We discuss a unilateral ovarian fibroma in a patient with Gorlin syndrome in the absence of any basal cell carcinoma skin lesions.

## **Case presentation**

**Figure 1:**
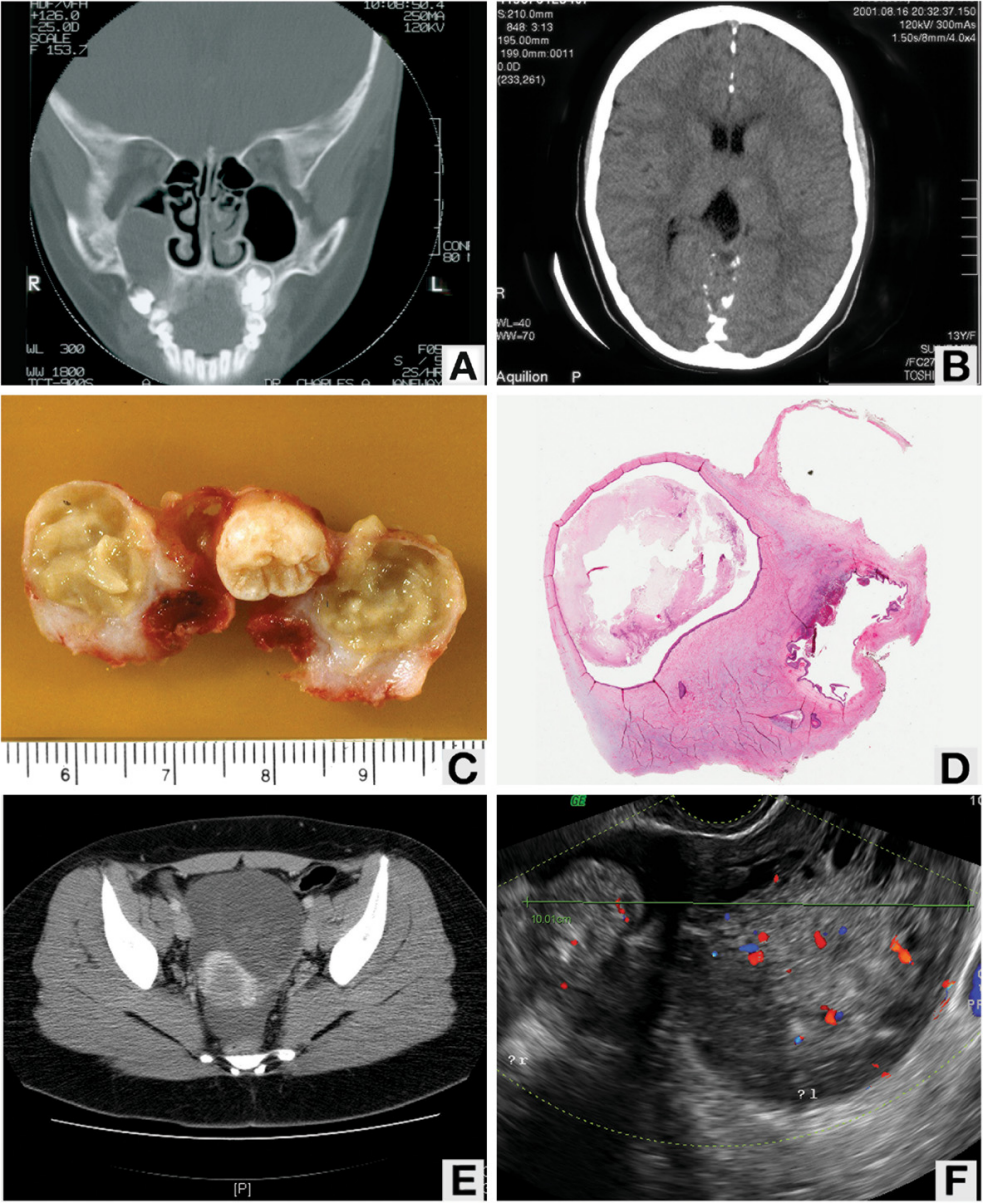
**Imaging studies. (A)** Computed tomography scan showing odontogenic keratocyst. **(B)** Computed tomography scan showing calcification of the falx cerebri. **(C)** Gross image of odontogenic keratocyst. **(D)** Photomicrograph of odontogenic keratocyst. **(E)** Computed tomography scan showing pelvic mass. **(F)** Ultrasound image showing pelvic mass.

Our patient was a Caucasian woman, 22 years of age and born by Cesarean section following an unremarkable gestation. She was described as having normal development and unremarkable schooling. Our patient had a history of recurrent dental infections and had multiple odontogenic keratocysts removed at ages 7, 13 and 20 from her maxilla and mandible (Figure 1A,C,D). At age 13, our patient was admitted to hospital for suspected viral meningitis. During this admission, a computed tomography scan of our patient’s head revealed calcification of her cerebral falx (Figure 1B). She required corrective lenses as a child and was diagnosed with a right optic nerve coloboma at the age of 13 years.

A diagnosis of Gorlin syndrome was initially suggested in 2009 when our patient was 20 years old, following the third excision of odontogenic keratocysts. At that time, investigations were also made for our patient’s irregular menses, with a pelvic ultrasound. The investigation revealed a 10 cm complex mass in the midline pelvis, suspected to be arising from the right adnexa (Figure 1F). The features of the mass were unclear and suggestive of ovarian neoplasm. An urgent computed tomography scan with intravenous contrast was performed, which demonstrated a multiloculated complex mass measuring 10.4 cm in largest dimension that represented either unilateral or bilateral ovarian masses (Figure 1E). There were solid and cystic areas with calcifications. The differential diagnosis at this point included a teratoma, other ovarian malignancies and chronic recurrent partial ovarian torsion. Our patient was scheduled for a right salpingo-oophorectomy with possible conversion to a bilateral salpingo-oophorectomy if there was intraoperative suspicion of malignancy. Given that she was nulliparous and desired to have children in the future, our patient was offered egg retrieval and embryo cryopreservation if the surgery was bilateral. Our patient underwent a successful right salpingo-oophorectomy with an intraoperative note of a 1 cm simple cyst of the left ovary and a Meckel’s diverticulum.

**Figure 2:**
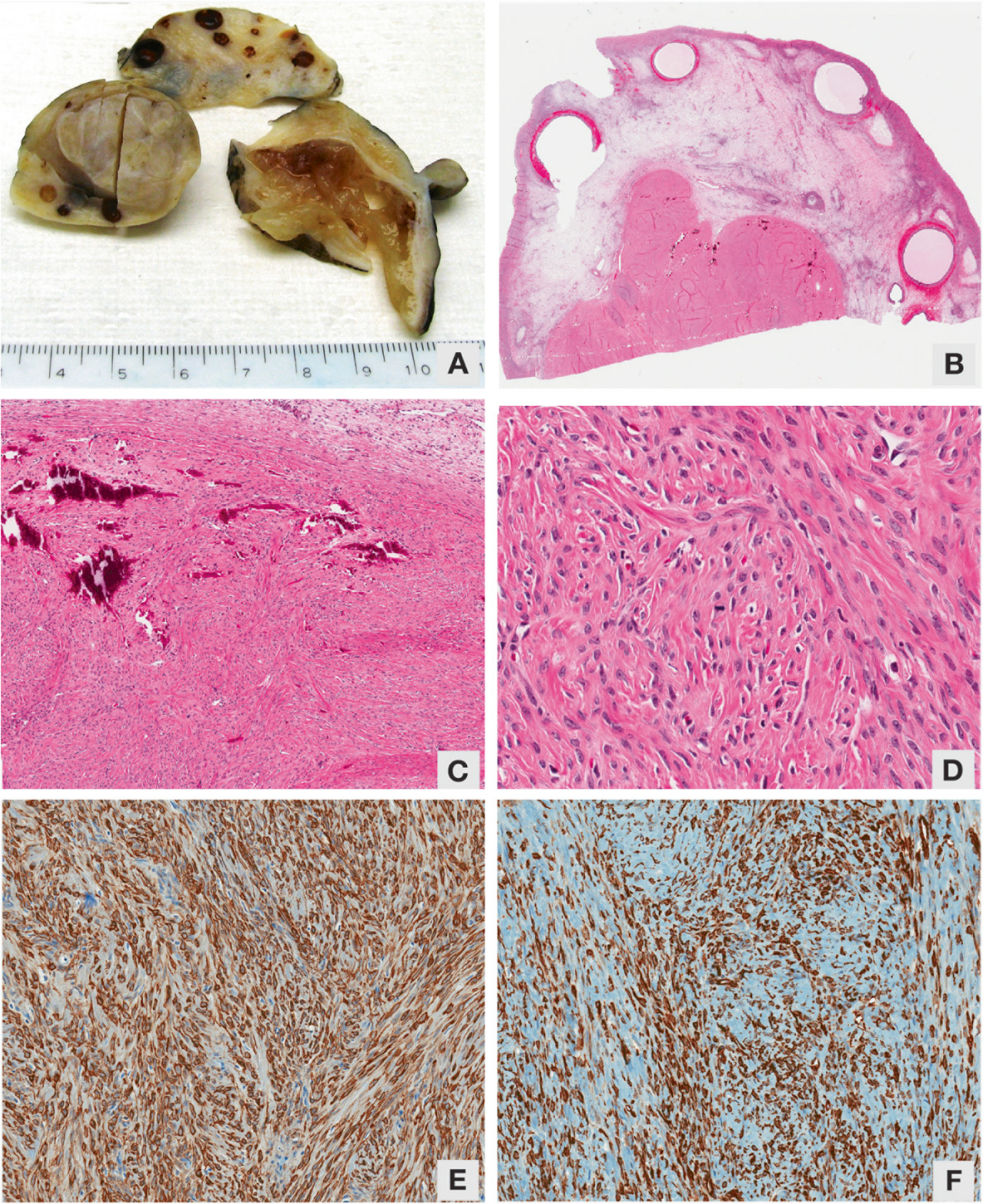
**Examination of the gross specimen. (A)** Gross image of ovarian fibroma. **(B)** Photomicrograph of ovarian fibroma. **(C)** Photomicrograph of ovarian fibroma showing calcifications. **(D)** Photomicrograph of ovarian fibroma, high power. **(E)** Smooth muscle actin positivity. **(F)** Vimentin positivity.

The gross specimen received from the right salpingo-oophorectomy consisted of a 12 × 10.5 × 7 cm cystic mass with a partial solid component measuring 4.5 × 4 × 3 cm (Figure 2A). An attached fallopian tube was identified measuring 7 × 0.7 × 0.6 cm. The cut surface of the cystic component of the mass revealed a diffuse gelatinous appearance. Approximately 10 mL of pale straw-colored fluid was extruded. The cut surface of the firm component revealed a partly solid white-tan whorled appearance with surrounding areas of suspected normal ovarian parenchyma.

On microscopic examination, the specimen demonstrated compressed ovarian tissue with secondary edema, follicular cysts and corpora lutea. The lesion was well-circumscribed with a variably cellular spindle cell proliferation arranged in intersecting bundles with abundant myxoid stroma. Areas of cystic degeneration and calcification were present. There was no cellular atypia and the mitotic rate did not exceed three per 10 high power fields (Figure 2B-D). Immunohistochemical staining was performed using an automated immunostainer (Ventana Benchmark XT, Ventana Medical Systems Inc., Tuscon, AZ, USA). Antibodies against smooth muscle actin (1A4, prediluted; Cell Marque, Rocklin, CA, USA), desmin (DE-R-11, prediluted; Ventana Medical Systems Inc.), vimentin (V9, prediluted; Ventana Medical Systems Inc.), calretinin (polyclonal, prediluted; Cell Marque), and inhibin (R1, 1:50; Dako North America Inc, Carpinteria, CA, USA) were used. Immunohistochemical staining of the lesion showed positivity for vimentin and smooth muscle actin, and negativity for inhibin, calretinin and desmin (Figure 2E,F).

## **Discussion**

Our case meets two major (odontogenic keratocysts of the jaw, bilamellar calcification of the falx cerebri) and one minor (ovarian fibroma) diagnostic criteria for Gorlin syndrome. Our patient did not have any basal cell carcinoma skin lesions, which typically develop between puberty and age 35 years and are most commonly located on the face, neck and upper trunk [1]. Our patient developed odontogenic keratocysts of the jaw from the age of 7 years. These lesions are commonly seen in Gorlin syndrome, developing usually after age 7 years and on average by 15 to 17 years, with 80% of patients typically being affected [1,3,6]. Most patients require more than three operations for removal of the keratocysts [3]. Calcification of the falx cerebri is reported to occur in 65% to 92% of individuals [3,5].

The ovarian fibroma in our case contained calcifications, which is a feature typically found in ovarian fibromas of Gorlin syndrome, but rarely in nonsyndromic cases [1]. Ovarian fibromas are reported in up to 75% of patients with Gorlin syndrome. Syndromic fibromas are also described as usually being multinodular and multifocal [10]. Our patient showed a lobular configuration that was unifocal. Ovarian fibromas associated with Gorlin syndrome are bilateral in 75% of cases [1], whereas in nonsyndromic cases they are bilateral in 8% of cases [10]. Large bilateral fibromas may overlap centrally and be confused with a calcified uterine fibroid [1]. In our case, the unilateral fibroma extended beyond our patient’s midline on imaging, which made it difficult to determine whether the lesion was unilateral or bilateral. The lesions are slow-growing tumors and generally are asymptomatic unless they are large enough to cause mass effects within the abdomen and pelvis, or if they undergo torsion [11].

On microscopy, the tumors are composed of tightly packed spindle cells with round to oval nuclei and have an intersecting bundle or storiform architecture, as is seen in our case. An edematous fibroma can be mistaken for massive ovarian edema or ovarian fibromatosis [10]. Typical fibromas contain a mitotic rate of three or less per 10 high power fields and no cellular atypia, which was also seen in our case. Ovarian fibromas in Gorlin syndrome are considered benign [2], although development of fibrosarcoma within existing bilateral ovarian fibromas has been reported [8]. Management involves conservative excision with an attempt at ovarian functional preservation. Ovarian fibromas rarely recur after surgery [11].

Techniques such as egg retrieval and embryo cryopreservation are useful for patients requiring complete bilateral oophorectomy and desiring future children, as was offered to the patient in our case. The patient should undergo genetic counseling because Gorlin syndrome is an autosomal dominant condition [12]. Children of patients with the syndrome should be investigated for clinical manifestations.

## **Conclusion**

We report the case of a patient with Gorlin syndrome who did not have any skin lesions and presented with a unilateral ovarian fibroma. Nevoid basal cell carcinoma syndrome, or Gorlin syndrome, is a rare multisystem disorder with multiple developmental anomalies and an increased risk of developing assorted benign and malignant tumors. Skin lesions are not necessary for the diagnosis of Gorlin syndrome and ovarian fibromas are typically bilateral in the syndrome. Ovarian fibromas are managed with surgical excision with an attempt at preserving ovarian function.

## **Consent**

Written informed consent was obtained from the patient for publication of this case report and accompanying images. A copy of the written consent is available for review by the Editor-in-Chief of this journal.

## **Competing interests**

The authors declare that they have no competing interests.

## **Authors’ contributions**

TF performed the histological examination of the ovary, and was the major contributor in writing the manuscript. CP performed the histological examination of the odontogenic keratocyst. KB performed the right salpingo-oophorectomy and followed our patient clinically. YEG performed the histological examination of the ovary. All authors read and approved the final manuscript.
